# *Porphyromonas gingivalis* Outer Membrane Vesicles Increase Vascular Permeability

**DOI:** 10.1177/0022034520943187

**Published:** 2020-07-29

**Authors:** C. Farrugia, G.P. Stafford, C. Murdoch

**Affiliations:** 1 School of Clinical Dentistry, University of Sheffield, Sheffield, UK

**Keywords:** endothelial cells, periodontal disease, cardiovascular disease, infection, zebrafish, vascular disease

## Abstract

Periodontitis is increasingly associated with increased risk of cardiovascular and other systemic diseases. The Gram-negative anaerobe, *Porphyromonas gingivalis*, is a key periodontal pathogen, and several lines of evidence link the presence of this bacterium in the circulation with vascular disease. The outer membrane vesicles (OMVs) produced by *P. gingivalis* have been shown to play a role in periodontitis, although, to date, little is known about their interaction with the vasculature; therefore, this study assessed the effects of *P. gingivalis* OMVs on the endothelium. OMVs were isolated from wild-type strain W83 and the gingipain-deficient strain ΔK/R-ab. Immunoblotting along with cryo-EM showed gingipain expression in W83 but not ΔK/R-ab-derived OMVs, where gingipains were localized to the cell wall surface. Confluent endothelial cell monolayers infected with either W83 or W83-derived OMV displayed significantly increased dextran permeability over those infected with ΔK/R-ab or its OMV. Moreover, W83-derived OMVs induced significantly more vascular disease in a zebrafish larvae systemic infection model over 72 h compared to those injected with gingipain-deficient OMVs or controls. In line with these data, human microvascular endothelial cells (HMEC-1) displayed an OMV-associated, gingipain-dependent decrease in cell surface levels of the intercellular adhesion molecule PECAM-1 (CD31) when examined by flow cytometry. These data show, for the first time, that OMVs from *P. gingivalis* mediate increased vascular permeability, leading to a diseased phenotype both in vitro and in vivo. Moreover, these data strongly implicate gingipains present on the OMV surface in mediating these vascular events, most likely via a mechanism that involves proteolytic cleavage of endothelial cell-cell adhesins such as PECAM-1. These data provide important evidence for the role of bacterial-derived OMVs in mediating systemic disease.

## Introduction

The association between periodontal disease and cardiovascular disease is well established. (Friedewald et al. 2009; Sanz et al. 2020). Periodontal disease has been found to increase the risk of both cardiovascular disease and coronary heart disease (Chhibber-Goel et al. 2016; Masi et al. 2019; Gustafsson et al. 2020). Nonetheless, the biological mechanisms through which this occurs are still unknown. Increasing evidence suggests that in cases of extreme gingivitis or periodontitis, the anaerobic periodontal pathogen, *Porphyromonas gingivalis*, can enter the bloodstream through inflamed and ulcerated periodontal tissue, an area coined the *porte d’entrée* (Loos 2005; Castillo et al. 2011). Here, loss of tissue integrity and increased bleeding facilitate movement of bacteria from the periodontal pocket into the bloodstream (Loos 2005; Schenkein and Loos 2013), with *P. gingivalis* repetitively detected in diseased vascular tissue (Kozarov et al. 2005; Gaetti-Jardim et al. 2009; Marcelino et al. 2010; Szulc et al. 2015), as well as disease-free femoral and coronary arteries (Mougeot et al. 2017).

*P. gingivalis* harbors several virulence factors that have been attributed to causing its pathogenic effects both locally and systemically. This includes gingipains, lysine, and arginine-specific cysteine proteases that cause virulence by their ability to cleave host proteins (Hočevar et al. 2018), not only avoiding immune response by degradation of cytokines and proinflammatory molecules (Nassar et al. 2002) but also mediating cell surface protein and extracellular matrix disruption, facilitating the loss of cellular and tissue integrity (Tada et al. 2003; Yun et al. 2005; Ruggiero et al. 2013). We recently showed that *P. gingivalis* dramatically increases the morbidity and mortality of zebrafish in a gingipain-dependent manner when injected systemically (Widziolek et al. 2016), suggesting that these proteases may play a key role in mediating vascular damage.

Like most Gram-negative organisms, *P. gingivalis* produces outer membrane vesicles (OMVs) that appear to retain many of the virulence factors of the parent cell, including lipopolysaccharide (LPS) (Haurat et al. 2011), fimbrae (Mantri et al. 2015), and gingipains (Haurat et al. 2011; Nakao et al. 2014). OMV-derived virulence factors have been shown to drive oral epithelial cell responses (Nakao et al. 2014; Cecil et al. 2016) and influence the differentiation and calcification of smooth muscle cells in vitro (Yang et al. 2016), suggesting that OMVs may affect cells of the vasculature. Interestingly, the presence of *P. gingivalis*–derived OMVs has been detected in the peripheral blood and cerebrospinal fluid in animal models with severe bacterial infections (Bai et al. 2015; Jia et al. 2015), indicating that OMVs may be widespread within the circulation and access areas of tissue not accessible to whole bacteria. However, the effects of OMVs on endothelial cells remain to be determined and require further research. Using an in vitro and in vivo approach, we show for the first time that *P. gingivalis*–derived OMVs significantly affect endothelial permeability in a gingipain-dependent manner, a process that may be mediated by cleavage of cell-to-cell adhesion molecules. These novel data demonstrate that *P. gingivalis* OMVs may play a pivotal role in disrupting the vasculature, a process that may drive or markedly increase the risk of cardiovascular disease.

## Materials and Methods

### Bacterial Culture and OMV Preparation

Wild-type *P. gingivalis* strain W83 and its isogenic gingipain-deficient mutant ΔK/R-ab (*kgp*Δ*598*-1732::*Tc^R^* *rgpA-::Cm^R^* *rgpB*Δ*410-507::Em^R^*; provided by Prof. Jan Potempa, Jagiellonian University, Kraków, Poland) were maintained on Fastidious Anaerobe agar (NeoGen) supplemented with 5% v/v oxalated horse blood and 1 µg/mL tetracycline. Bacteria were inoculated into brain-heart infusion broth (Oxoid) containing 5 mg/mL yeast extract, 250 µg/mL L-cysteine, 1 mg/mL hemin, and 1 mg/mL vitamin K and incubated anaerobically (37°C, 80% N_2_, 10% CO_2_, and 10% H_2_). For OMV isolation, freshly grown bacterial cultures (OD_600_ = 1, equivalent to 9 × 10^9^ colony-forming units [CFUs]) were centrifuged (8,000 g, 4°C, 5 min) and the pellet collected. The supernatant was filtered (0.2 µm) and further centrifuged for 1 h at 100,000 g, 4°C. The resulting OMV pellet was washed once with phosphate-buffered saline (PBS), ultracentrifuged again, resuspended in PBS, and characterized using nanoparticle-tracking analysis (ZetaView).

### Immunoblot Analysis

Protein concentrations of bacterial cell pellets and OMV were measured by a bicinchoninic acid (BCA) protein assay. Samples (10 µg protein) were run on 4% to 12% NuPAGE gels, transferred to nitrocellulose membranes, and then blocked with 5% w/v milk protein in Tris-buffered saline (TBS). Following washing with TBS–Tween-20 (0.1%), membranes were incubated with either rabbit Rb7 antiserum (Aduse-Opoku et al. 2006) or mouse monoclonal antibody 1B5 (Curtis et al. 1999) (gifts from Professor Mike Curtis, King’s College London, London, UK). Immunoreactive bands were visualized using horseradish peroxidase–conjugated IgG antibody followed by ECL substrate (Thermo Scientific).

### Immunogold Cryo–Electron Microscopy

Immunogold cryo–electron microscopy (EM) was performed as described by Chen et al. (2011) with modifications. Briefly, exponential phase-grown W83 and ΔK/R-ab were adjusted to OD_600_ = 1, pelleted by centrifugation (10 min, 6,000 g at 10°C), washed, and resuspended in PBS. Cell suspensions were blocked with 3% bovine serum albumin (BSA) at 4°C, then incubated with MAb 1B5 (1/100 dilution) in 1% BSA for 1 h. After washing, cells were incubated with 12 nm gold-conjugated goat anti-mouse antibody (Abcam; 1/20 dilution) for 1 h and then washed with PBS. For cryo-EM, a 5-µL sample was applied onto a Quantifoil R3.5/1 holey carbon film mounted on a 300-mesh copper grid (Quantifoil MicroTools GmbH), rendered hydrophilic by glow discharge in a reduced atmosphere of air for 30 s. The grid was then frozen in liquid ethane and imaged under cryogenic temperatures using a Tecnai Artica (FEI Co.) at 200 kV, equipped with a Falcon 3 Camera (Gatan). Micrographs were recorded under low-dose conditions with underfocus values of 4 to 10 µm.

### Gingipain Activity

Arg- and Lys-gingipain proteinase activity was determined using a fluorescence-based substrate activity assay as described previously (Naylor et al. 2017). For Arg-proteinase activity, 100 µL PBS containing 1 mM L-cysteine and 200 µM αN-benzoyl-L-arginine-7-amido-4-methylcourmarin was added to 50 µL (4.5 × 10^8^ CFUs) of each sample. Lys-proteinase activity was quantified using 100 µL PBS containing 1 mM L-cysteine, 10 µM D-ab-Leu-Lys-7-amido-4-methylcourmain, and 50 µL of sample. After a 10-min incubation, the reaction was terminated by the addition of 200 µM or 500 µM N-α-tosyl-L-phenylalanine chloromethyl ketone for Arg and Lys activity, respectively. In both assays, released 7-amido-4-methylcourmarin was measured spectrophotometrically at a 365-nm excitation and a 460-nm emission.

### Cell Culture, Infection, and Flow Cytometry

Immortalized human microvascular endothelial cells (HMEC-1) (Ades et al. 1992) were grown in MCDB131 supplemented with 10 ng/mL epidermal growth factor, 1 µg/mL hydrocortisone, 10% fetal calf serum, and 2 mM L-glutamine. For flow cytometry, confluent HMEC-1 cultured in 6-well plates were infected with W83, ΔK/R-ab (multiplicity of infection [MOI] of 100), or OMVs (2.8 × 10^10^ particles/mL) derived from these bacteria for 1.5 h at 37°C in serum-free medium. For inhibition of gingipain activity, OMVs were pretreated with 2 µM KYT-1 and KYT-36 for 30 min in anaerobic conditions prior to isolation. Medium alone was used as control. Following infection, HMEC-1 were washed, removed from plates using 0.02% ethylenediaminetetraacetic acid for 20 min, and resuspended in 100 µL FACS buffer (0.1% BSA, 0.1% sodium azide in PBS). Phycoerythrin-Cyanine7-conjugated anti-human CD31 (clone MW59) or isotype-conjugated IgG control was added for 45 min on ice. Cells were washed and resuspended in FACS buffer and analyzed using a LSRII flow cytometer (BD Biosciences). FlowJo software (TreeStar) was used to calculate the normalized median fluorescence index (nMFI).

### Fluorescent Dextran Permeability Assay

A fluorescent dextran permeability assay was performed as previously described (Wang and Alexander 2011). Fibronectin-coated (10 µg/mL) 0.4-µm pore, hanging cell culture inserts (Millicell) were seeded with HMEC-1 until confluent and then incubated with W83 or ΔK/R-ab whole cells (MOI 100) or OMVs (2.8 × 10^10^ particles/mL) derived from these bacteria for 1.5 h at 37°C in serum-free medium. Inserts without cells or HMEC-1 alone were used as controls. Solutions were removed, inserts were transferred to a new plate containing 500 µL supplemented MCDB131, and 450 µL supplemented MCDB131 containing 65 µg/mL 70 kDa fluorescent dextran (Molecular Probes) was added to the insert. Dextran leakage through the cell monolayer to the bottom well was monitored hourly for a 5-h period by aspirating 250 µL medium from the bottom well and measuring dextran fluorescence at a 494-nm excitation and 521-nm emission. The aspirated volume was replaced with supplemented MCDB131 for further readings.

### Systemic Injection into Zebrafish Larvae

Zebrafish maintenance and experimental work was performed in accordance with UK Home Office regulations and the UK Animals (Scientific Procedures) Act of 1986 and under project license P1A4A7A5E using larvae under 5 d postfertilization (dpf). London wild-type inbred zebrafish larvae were maintained in E3 medium at 29°C according to standard protocols. The 30-h postfertilization (hpf), Tricaine-anesthetized, dechorionated zebrafish larvae were injected with PBS, 5 × 10^4^ CFU W83, or OMVs (1.15 × 10^5^ particles) derived from W83 or ΔK/R-ab via direct systemic inoculation into the common cardinal vein (Widziolek et al. 2016). Zebrafish viability was assessed by examining the presence of a heartbeat and blood flow within the circulation. Live imaging was performed using a stereomicroscope (WILD) equipped with a camera.

### Statistical Analysis

Data are presented as mean ± standard deviation (SD) from at least 3 independent experiments carried out in triplicate except for flow cytometry data where nMFI was used. Differences between 2 groups were assessed using either Student’s *t* test or Mann-Whitney *U* test, while differences between group data were assessed using 1-way analysis of variance (ANOVA) followed by Tukey’s post hoc multiple comparison test for parametric or nonparametric data, respectively, following a normality test. Survival data were evaluated using the Kaplan-Meier method, and comparisons between individual curves were made using the log-rank test. Statistical analysis was performed using GraphPad Prism v8.4.0 (GraphPad Software), and statistical significance was assumed at *P* < 0.05.

## Results

### Characterization of Wild-Type W83 and ΔK/R-ab-Derived OMVs

**Figure 1.**
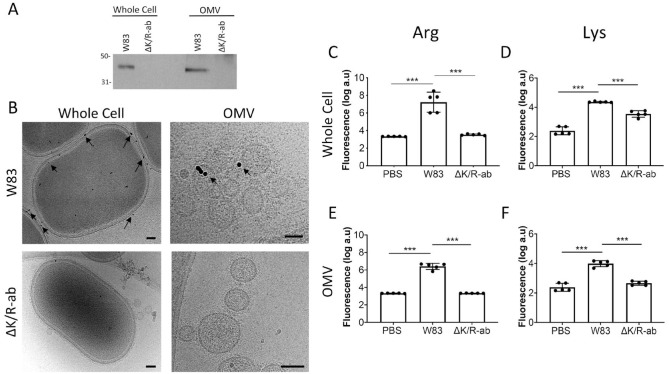
Presence and activity of wild-type and ΔK/R-ab *Porphyromonas gingivalis* gingipains on whole bacteria and outer membrane vesicles (OMVs). (**A**) Immunopositive bands of 45 kDa were observed in the W83 whole-cell and OMV samples but not in the gingipain-null ΔK/R-ab equivalents when protein extracts were analyzed by immunoblotting using the Rb7 antigingipain antiserum. (**B**) Cryo–electron microscopy (EM) micrographs showing mAb 1B5 immunogold-labeled W83 bacteria and OMV. The gingipain expression is mainly located to the cell wall in both W83 whole cells and OMVs (black arrows) but is absent in ΔK/R-ab equivalents (scale bar: whole bacteria = 100 nm; OMV = 50 nm). (**C–F**) Gingipain fluorometric enzyme activity assays showing the higher levels of activity of arginine-specific (Arg, C, E) and lysine-specific (Lys, D, F) protease in W83 whole cells and OMVs compared to ΔK/R-ab mutant equivalents. In C–F, data are mean ± SD of 5 independent experiments with each individual experiment performed in triplicate. Statistical significance was determined by 1-way analysis of variance, ***P < 0.001.

Our aim was to investigate whether *P. gingivalis* OMVs and their associated gingipains might mediate endothelial damage. We first characterized OMVs from wild-type W83 and its isogenic gingipain-negative (ΔK/R-ab) strain. Nanoparticle analysis showed that numbers of OMVs produced from these 2 strains were comparable (Appendix Fig. 1A). Overall, wild-type W83 OMVs were 24% larger in size (*P* < 0.05, Appendix Fig. 1B, C) than those from ΔK/R-ab (144 ± 23 nm). As expected, immunoblotting with the RgpA/B-specific antisera Rb7 produced immunoreactive bands of 45 kDa for whole W83 cells and W83-derived OMVs, whereas this band was absent from counterpart ΔK/R-ab samples (Fig. 1A). Similar immunoblot data were obtained when using the monoclonal antibody 1B5 that binds to a shared glycan epitope between Rgp and the minority A-LPS of *P. gingivalis* (Appendix Fig. 2). Furthermore, the presence of gingipains on the bacterial surface and periphery of purified W83-derived OMVs was confirmed by cryo-EM using 1B5 monoclonal antibody immunogold labeling, whereas ΔK/R-ab OMV did not show any immunoreactivity (Fig. 1B). Cryo-EM did not reveal any morphological differences between whole cells and OMVs from either strain (Fig. 1B). Finally, W83 whole cells and OMVs displayed the expected gingipain enzyme activity for both lysine- and arginine-based substrate that was absent in the ΔK/R-ab strain (*P* < 0.001; Fig. 1C–F).

### Gingipains Mediate Increased Endothelium Permeability In Vitro

**Figure 2.**
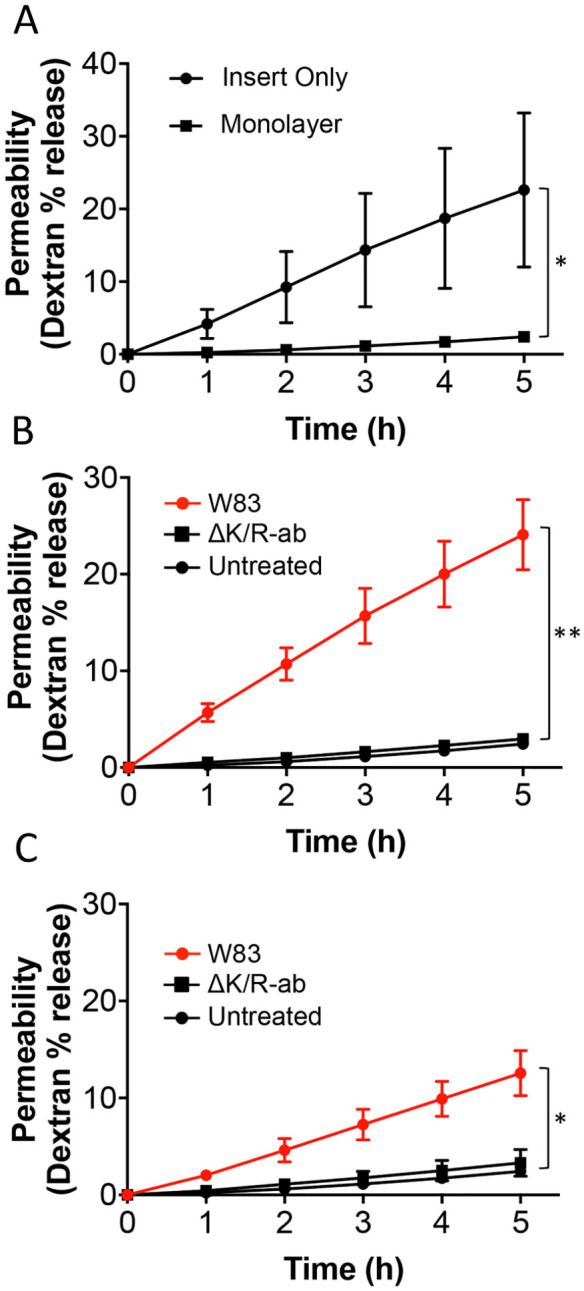
Increased endothelium permeability in vitro following treatment with *Porphyromonas gingivalis* whole cells and outer membrane vesicles (OMVs) is gingipain dependent. (**A**) Movement of fluorescently labeled 70 kDa dextran from the upper well to the lower well in a Transwell assay increased in a time-dependent manner in the absence of human microvascular endothelial cells (HMEC-1; insert only), whereas this movement was almost abolished when a confluent endothelium was cultured on the insert surface (monolayer). Endothelial monolayers were treated with (**B**) whole bacteria or (**C**) OMVs from either W83 or ΔK/R-ab for 1.5 h, and then dextran permeability across the endothelium was measured for up to 5 h; phosphate-buffered saline (PBS)–treated endothelium was used as controls. Increased endothelial permeability was significantly increased in a time-dependent manner following exposure to W83 when compared to ΔK/R-ab equivalents and untreated controls for both whole bacteria and OMVs. No significant differences were observed between ΔK/R-ab-treated and uninfected controls. Data are presented as mean ± SD of 3 independent experiments and were analyzed by 1-way analysis of variance followed by Tukey’s post hoc multiple comparisons test. *P < 0.05. **P < 0.01.

Since increased vascular permeability has been linked to cardiovascular risk (Chistiakov et al. 2015), we performed a fluorescent dextran-based in vitro permeability assay on confluent HMEC-1 monolayers to determine the influence of OMV-expressed gingipains on endothelial permeability. Confluent, untreated endothelial monolayers proved an effective barrier with little of the applied 70-kDa fluorescent dextran permeating the cell layer after 5 h (Fig. 2A). In contrast, the endothelium displayed significantly increased dextran permeability upon treatment with whole-cell W83 (Fig. 2B; *P* < 0.01) or W83-dervied OMVs (Fig. 2C; *P* < 0.05) compared to counterpart ΔK/R-ab-treated or noninfected controls, suggesting that altered vascular permeability is gingipain dependent. Notably, endothelium permeability was significantly higher (*P* < 0.05) in the presence of W83 whole cells (4.8% dextran/h) compared to OMVs (2.5% dextran/h).

### OMV-Associated Gingipains Are Responsible for Systemic Symptoms in a Zebrafish Larvae Infection Model

**Figure 3.**
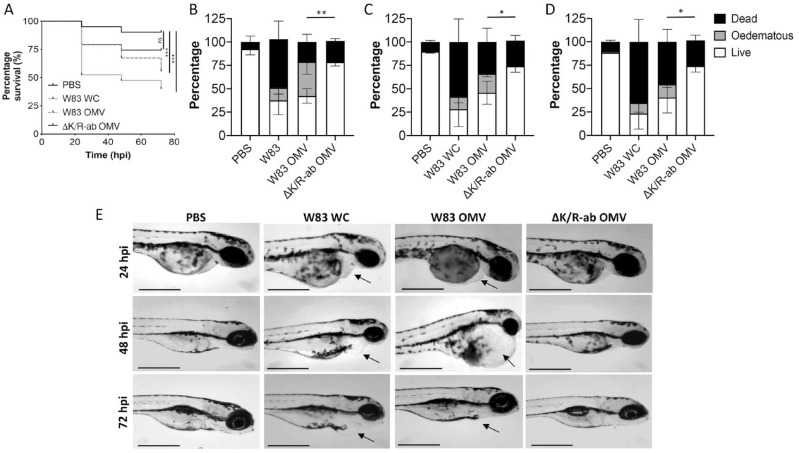
W83 outer membrane vesicles (OMVs) induce systemic disease in zebrafish larvae in a gingipain-dependent manner. (**A**) Kaplan-Meier survival plots of zebrafish larvae infected 30-h postfertilization (hpf) with phosphate-buffered saline (PBS) control, *Porphyromonas gingivalis* (*Pg*) W83 whole cells (WCs), *Pg* W83 OMVs, or ΔK/R-ab OMVs. Comparison of survival curves using the log-rank test shows significant differences between W83 whole cell–injected and W83 OMV-injected zebrafish compared to PBS controls. Survival curves of zebrafish larvae injected with ΔK/Ra-b OMVs were not statistically different from the PBS control (ns = no significant difference, ***P < 0.001). (**B–D**) Percentage live, edematous, and dead zebrafish larvae at (B) 24, (C) 48, and (D) 72 hpi showing that the percentage of diseased (dead + edematous) zebrafish was significantly increased following systemic infection with W83 OMVs compared to ΔK/R-ab OMVs at all time points (*P < 0.05, **P < 0.01 by 1-way analysis of variance with Tukey’s post hoc multiple comparisons test). (**E**) Representative micrographs showing the morphology of zebrafish larvae infected with PBS control, W83 whole cells (WCs), W83 OMVs, or ΔK/R-ab OMVs. W83 whole-cell and OMV-infected zebrafish showed marked edema around yolk sac and heart (black arrows). Scale bars = 500 µm. Data in A–D are mean ± SD pooled from 3 independent experiments with at least 39 zebrafish total per group.

**Figure 4.**
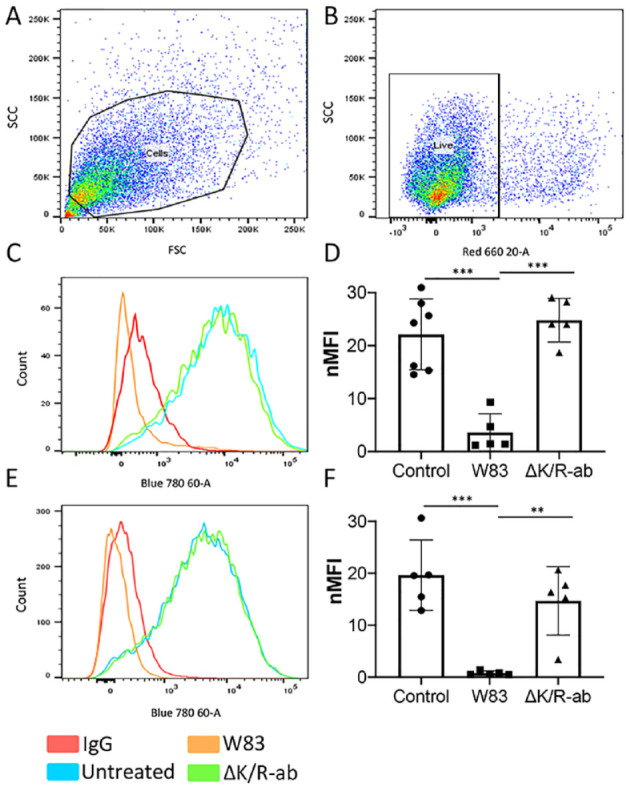
Loss of endothelial cell surface PECAM-1 by W83 whole bacteria and outer membrane vesicles (OMVs) is mediated by gingipains. Following a 1.5-h exposure to W83 or ΔK/R-ab whole bacteria or OMVs, human microvascular endothelial cells (HMEC-1) were removed from tissue culture plates and subjected to flow cytometric analysis for PECAM-1 cell surface abundance. Cells were gated using (**A**) side-scatter (SSC) and forward-scatter (FSC) voltages, then for (**B**) cell viability using TO-PRO-3 live/dead staining. Untreated cells were used as controls. Representative histograms and bar chart of 10,000 gated cells showing that PECAM-1 cell surface abundance is significantly decreased upon treatment with (**C, D**) W83 whole bacteria and (**E, F**) W83-derived OMVs compared to ΔK/R-ab-treated equivalents and untreated controls. PECAM-1 cell surface abundance was similar on HMEC-1 treated with ΔK/R-ab whole bacteria or ΔK/R-ab-derived OMVs to those observed on untreated controls. Data in D and F are presented as mean ± SD normalized median fluorescence index (nMFI) from 5 independent experiments with statistical significance determined by a 1-way analysis of variance with Tukey’s post hoc multiple comparisons test. **P < 0.01. ***P < 0.001.

We have previously shown that zebrafish larvae display increased mortality (death) and morbidity (cardiac and yolk edema) when systemically injected with *P. gingivalis* (Widziolek et al. 2016). The presence of gingipain on the surface of OMV suggests that these may contribute to systemic disease. Kaplan-Meier survival plot analysis showed that both whole-cell W83 and W83-derived OMVs caused significantly more zebrafish mortality than PBS-injected controls (*P* < 0.001; Fig. 3A). In contrast, morbidity in zebrafish larvae injected with ΔK/R-ab-derived OMVs was not significantly different from controls. To interrogate the OMV data further, we stratified the fish into viable or diseased (nonviable + edematous) groups. A significant increase in the number of diseased zebrafish treated with W83-derived OMVs was observed when compared to ΔK/R-ab-derived OMVs in a time-dependent manner (Fig. 3B–D). W83 OMV-treated zebrafish larvae displayed marked cardiac edema and enlarged yolk sack, whereas those injected with ΔK/R-ab-derived OMV or PBS-treated controls displayed mild or no edema (Fig. 4E), providing further evidence that gingipains present on the surface of OMVs can cause systemic disease in vivo.

### OMV-Expressing Gingipains Cleave Endothelial Cell Adhesion Molecules

**Figure 5.**
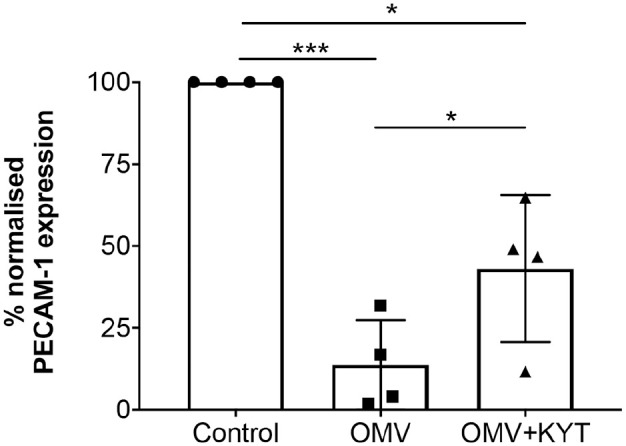
Inhibition of gingipain activity prevents ablation of PECAM-1 expression following W83 outer membrane vesicle (OMV) infection. W83 OMVs were treated with 2 µM KYT gingipain inhibitors for 1 h prior to human microvascular endothelial cell (HMEC-1) infection. HMEC-1 treated with W83 OMVs or untreated cells were used as controls. Flow cytometric analysis showed that the gingipain-specific inhibitor, KYT, prevented the loss of PECAM-1 cell surface abundance that was mediated by W83 OMVs. Data are mean ± SD normalized median fluorescence index (nMFI) from 4 independent experiments with statistical significance determined by a 1-way analysis of variance with Tukey’s post hoc multiple comparisons test. *P < 0.05. ***P < 0.001.

PECAM-1 is a major endothelial adhesion molecule responsible for maintaining vascular integrity at cell-cell junctions, with its loss leading to increased vascular leakage (Privratsky and Newman 2014). Previous studies have shown that gingipains can cleave recombinant PECAM-1 (Yun et al. 2005; Sheets et al. 2006; Widziolek et al. 2016). We therefore examined if OMV-associated gingipains could cleave intercellular PECAM-1. Treatment of HMEC-1 monolayers with W83 or W83 OMVs did not alter endothelial viability (Fig. 4A, B). In contrast, PECAM-1 cell surface abundance was significantly (*P* < 0.001) decreased following infection with whole-cell W83 (Fig. 4C, D) and W83-derived OMVs (Fig. 4E, F) compared to both untreated controls and the ΔK/R-ab equivalents. To confirm these findings, W83-derived OMVs were pretreated with the gingipain-specific protease inhibitors KYT1 and KYT36 before incubation with HMEC-1 monolayers. Here, inhibition of gingipain activity significantly (*P* < 0.05) prevented OMV-mediated cleavage of PECAM-1 (Fig. 5), indicating that loss of cell surface PECAM-1 by W83 OMV is gingipain mediated.

## Discussion

Periodontal disease is one of the most common diseases worldwide and a major public health issue (Tonetti et al. 2017). It is frequently associated with several systemic conditions, leading to the notion of the now commonly phrased “oral health systemic connection” (Tonetti et al. 2013). Like many bacteria,*P. gingivalis* produces abundant OMVs (Xie 2015), although there are limited data as to their effects in host-pathogen interactions. Here, we show for the first time that *P. gingivalis* OMVs dramatically increase vascular permeability in vitro and potentiate vascular edema and mortality in vivo in a gingipain-dependent manner, suggesting that they may act in concert with whole bacteria to affect cardiovascular disease risk.

Gingipains are key virulence factors of *P. gingivalis*. As well as functions in bacterial coaggregation, biofilm formation, and heme acquisition, they also cleave soluble and cell surface human proteins (Hočevar et al. 2018). Since both RgpA/B and Kgp gingipains have been previously detected in *P. gingivalis–*derived OMVs by mass spectrometry (Haurat et al. 2011), we reasoned that gingipain-expressing OMVs might be a key mediator of endothelial cell surface receptor degradation, leading to increased vascular permeability. This may be important in the context of systemic disease as their small size and abundance are likely to allow OMVs to penetrate host tissue micro-niches that may not be readily accessible to *P. gingivalis* whole cells. To test our hypothesis, we generated OMVs from wild-type W83 and its isogenic gingipain-deficient counterpart, ΔK/R-ab, and confirmed presence or absence of gingipains on these strains/OMVs as previously observed using W50 and other *P. gingivalis* strains (Curtis et al. 1999; Aduse-Opoku et al. 2006; Naylor et al. 2017). Immunogold labeling followed by cryo-EM also showed that gingipains were located to the OMV cell surface. Although no structural abnormalities were visibly observed by cryo-EM, nanoparticle-tracking analysis showed that W83-derived OMV were larger in size than their gingipain-deficient counterparts. It is plausible that this size difference is due to changes in the molecular structure within the cell wall owing to loss of gingipain-mediated cell wall processing.

Very few studies have examined the role of *P. gingivalis* OMVs on vascular biology. Bartruff et al. (2005) showed that *P. gingivalis* ATCC33277-derived OMVs inhibited human umbilical vein endothelial cell (HUVEC) proliferation by up to 80% as well as capillary tubule formation in an OMV dose-dependent manner. These effects were inhibited by heat treatment but not by protease inhibitors, suggesting that these effects were protein but not protease mediated, although no specific factor was identified (Bartruff et al. 2005). Using the same *P. gingivalis* strain, Jia et al. (2015) observed that OMVs suppressed endothelial nitric oxide synthase (eNOS) transcript and protein expression in HUVECs via activation of the ERK1/2 and p38 MAPK signaling pathways in a Rho-associated protein kinase-dependent manner. This study provides good evidence that OMVs may regulate vascular oxidative injury, although the OMV factors driving this effect were not examined. *P. gingivalis*–derived OMVs have recently been shown to promote vascular smooth muscle cell differentiation and calcification by increasing the activity of runt-related transcription factor 2 that is crucial in driving osteoblastic differentiation and mineralization of vascular smooth muscle cells (Yang et al. 2016).

Our study provides further evidence that OMVs can significantly perturb endothelial homeostasis. In vitro, W83-derived OMVs not only cleaved PECAM-1 on endothelial cell (HMEC-1) monolayers but also increased their permeability. Moreover, cleavage of PECAM-1 was significantly reduced when W83-derived OMVs were either preincubated with the gingipain protease inhibitors KYT1 and KYT36 or infected with gingipain-deficient OMVs. Not only do these data show that*P. gingivalis* OMVs mediate vascular damage but also that this is via a gingipain-dependent mechanism, the first time that this has been documented for *P. gingivalis* OMVs. We confirmed some of these in vitro observations in vivo using a systemic zebrafish infection model. Although zebrafish larvae have been used extensively to examine systemic host-pathogen interactions (Sullivan et al. 2017), only a few studies have examined the role of bacterial OMVs in systemic disease, and to our knowledge, none have been performed using *P. gingivalis* OMVs. OMVs derived from W83 but not ΔK/R-ab caused significant edema and mortality in zebrafish larvae, although the effects were less extreme than those observed with injection of whole-cell W83. These in vitro and in vivo data further confirm that OMVs have the potential to cause disease in the absence of whole-cell bacteria from which they are derived and augment current evidence that OMVs are able to exert their effects beyond that of the periodontal pocket.

Our data lead to the speculation that gingipains on OMVs as well as whole bacteria cleave endothelial intercellular junction proteins such as PECAM-1 and likely other adhesion molecules (VE-cadherin, CD99), thereby loosening cell-to-cell contacts to permit increased endothelial cell permeability. This may have 2 consequences: first, to allow exudate from the circulation into tissues leading to tissue edema, which we observed in vivo, and, second, to expose underlying connective tissue that may lead to platelet activation and subsequently foci for immune cell activation on the endothelium that would have dramatic implications for increased risk of systemic disease (Chistiakov et al. 2015). Moreover, the nanoscale size of OMVs would allow proteolytic damage to occur at vascular sites not accessible to whole bacteria. Although this hypothesis requires further evaluation, our data provide a potential mechanism for the link between periodontal disease and cardiovascular disease. It also provides clear evidence that the role of OMVs in host-microbial pathogenesis may be as important as whole bacteria, a factor that needs to be taken into consideration in the ongoing drive to decipher the oral health systemic connection.

## Supplemental Material

DS_10.1177_0022034520943187 – Supplemental material for *Porphyromonas gingivalis* Outer Membrane Vesicles Increase Vascular Permeability Click here for additional data file. Supplemental material, DS_10.1177_0022034520943187 for *Porphyromonas gingivalis* Outer Membrane Vesicles Increase Vascular Permeability by C. Farrugia, G.P. Stafford and C. Murdoch in Journal of Dental Research
