# Supplementary material for: Porphyromonas gingivalis Outer Membrane Vesicles Increase Vascular Permeability
Source: J Dent Res. 2020 Jul 29;99(13):1494–501. doi: 10.1177/0022034520943187 (PMC7684789; doi:10.1177/0022034520943187)
Supplement: DS_10.1177_0022034520943187 – Supplemental material for Porphyromonas gingivalis Outer Membrane Vesicles Increase Vascular Permeability [file DS_10.1177_0022034520943187.pdf]

P. gingivalis outer membrane vesicles increase vascular permeability

Cher Farrugia, Graham P. Stafford, Craig Murdoch

School of Clinical Dentistry, University of Sheffield, Sheffield, S10 2TA, UK

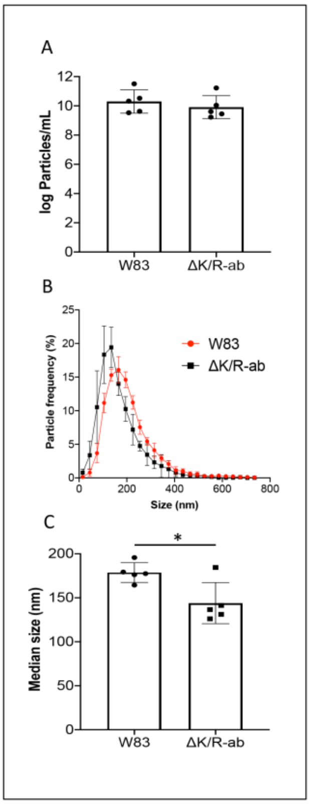

Appendix Figure 1: OMV quantification and particle size profile

ZetaView nanoparticle-tracking analysis of *P. gingivalis*-derived OMV from wild-type W83 and its isogenic gingipain-deficient mutant, ΔK/R-ab, following overnight culture and isolation by ultracentrifugation. (**A**) The number of OMVs released were similar for both W83 and ΔK/R-ab. (**B**) nanoparticle size distribution histogram and **(C)** quantification of median particle size analysis for W83 and ΔK/R-ab-derived OMVs. For ZetaView analysis, instrument calibration was performed using 100 nm polystyrene beads. Samples were proportionately diluted in PBS and nanoparticle tracking measurements recorded through three cycles of readings at 11 different positions. Data in all panels are means ± SD of 5 independent experiments and statistical significance was determined using Students *t* test, *p<0.05.

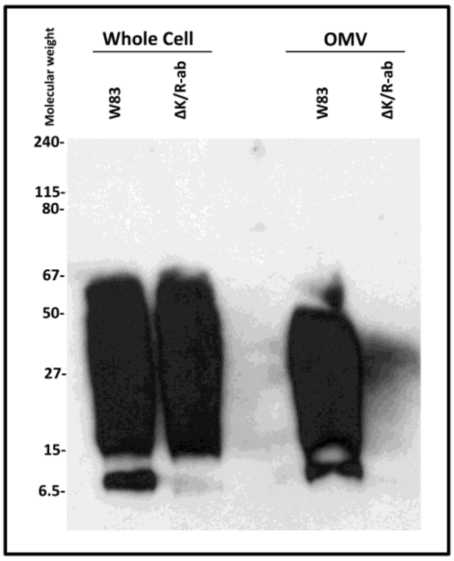

Appendix Figure 2: Gingipain expression on whole cell and OMV from W83 and ΔK/R-ab using monoclonal antibody 1B5. Protein samples from W83 and ΔK/R-ab whole bacteria as well as W83-derived and ΔK/R-ab-derived OMVs were separated on 4-12% NuPAGE® gels, transferred to nitrocellulose membranes and immunoblotted against mouse monoclonal antibody 1B5, an antibody that binds to the shared glycan epitope between Rgp and A-lipopolysaccharide of *P. gingivalis* (observed in the ΔK/R-ab whole cell lane and as a much weaker band in ΔK/R-ab OMVs). Banding patterns are as observed previously described (Aduse-Opoku et al. 2006; Curtis et al. 1999).

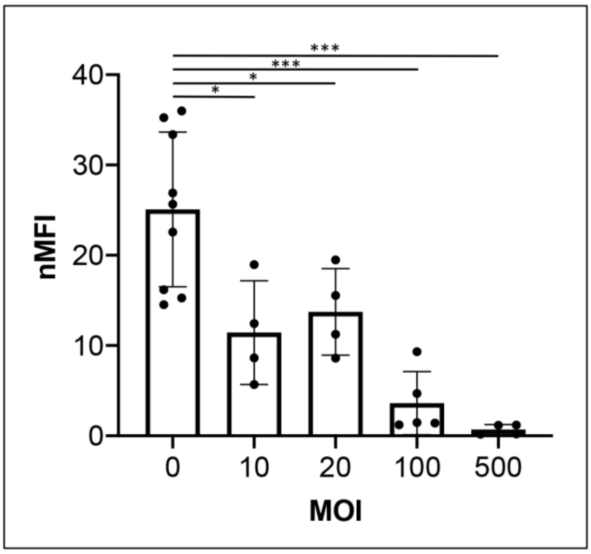

Appendix Figure 3: Reduced PECAM-1 cell surface abundance on HMEC-1 by *P. gingivalis* strain W83 is dose-dependent. HMEC-1 cells were infected with increasing MOI for 1.5 h and then cell surface abundance of PECAM-1 assessed by flow cytometry. Uninfected cells were used as controls. The reduction in PECAM-1 abundance was dose-dependent. Flow cytometric data are displayed as normalized median fluorescence intensity (nMFI). Statistical differences were analysed by 1-way ANOVA with Tukey's multiple comparison test *p<0.05, ***p<0.001 compared to uninfected control.
